# Porous carbons with complex 3D geometries via selective laser sintering of whey powder

**DOI:** 10.1038/s41598-024-84976-y

**Published:** 2025-01-13

**Authors:** Raúl Llamas-Unzueta, Alejandro Reguera-García, Miguel A. Montes-Morán, J. Angel Menéndez

**Affiliations:** Instituto de Ciencia y Tecnología del Carbono, INCAR-CSIC, c/Francisco Pintado Fe 26, Oviedo, 33011 Spain

**Keywords:** Engineering, Materials science

## Abstract

In addition to the inherent limitations of carbons to melt or flow, a vast majority of carbon precursors deforms during carbonisation, with stereolithography of thermoset resins being the preferred technology for 3D printing of carbons. An alternative is now presented with the possibility of using a melting-based technology, selective laser sintering (SLS), to fabricate 3D structures that withstand carbonisation. The key factor that makes this happen is whey powder, a natural, abundant and cheap by-product of the dairy industry. When heating the whey powder with a laser at 180–200 ºC for a few seconds, whey particles sinter, and 3D structures are obtained layer-by-layer. Carbonisation of the sintered whey structures brings about 3D porous carbons with excellent mechanical properties that preserve the SLS printed form albeit an isotropic shrinkage (approx. 23%). Melanoidins are identified as responsible for both the sintering and the thermoset behaviour during carbonisation of the whey powder.

**Supplementary Information:**

The online version contains supplementary material available at 10.1038/s41598-024-84976-y.

## Introduction

Additive manufacturing (AM) is having an impact on a number of applications, from analytical chemistry to biomedicine, electrochemistry and chemical reactors, due to its ability to provide materials with bespoke 3D structures^1–3^. There is a continuous quest for materials to be processed using the multiple AM technologies available, with polymers and metals showing a consolidated hegemony in the field^4^. Carbons, on the other hand, are a wide family of materials with unrivalled properties in terms of electrical and thermal conductivities, porosity, chemical inertness and mechanical performance. Carbons (including nanocarbons) have found a niche in AM mainly as fillers in polymeric formulations, either as fibres or particles, or as the active component in inks, with potential applications in aerospace, building, medical, catalysis, energy, among many other sectors^5^. Yet the possibility of using a conventional route in which a carbon precursor could be 3D printed and then carbonised to obtain a carbon material is hampered by the inherent limitation of most of the AM technologies, namely the need for the printed material (i.e., the carbon precursor) to flow or melt^5^.

3D printing of thermoset carbon precursors rather than thermoplastics is, in principle, required to obtain structures that withstand carbonisation. Stereolithography (SLA) based techniques are preferred for 3D printing thermosetting photopolymers^6^, and few works have already explored the possibility of carbonising the resulting structures^7–11^. In spite of the unbeatable resolution that SLA-based technology provides, only photocurable resins with very specific formulations render 3D structures that preserve their geometry upon direct carbonisation. Alternatively, other SLA printed resins require a tedious post-processing involving impregnation with different chemicals to avoid the collapse of the structure during the heat treatment^12,13^. In addition to SLA, AM technologies such as Direct Ink Writing (DIW) and Binder Jetting has also been used to print carbon precursors that were subsequently carbonised^14–18^. DIW printing of thermoset polymers is however not straightforward^19,20^, and the results obtained so far have had limited success in terms of dimensions and printing precision of the final carbon 3D structures^14–16^.

Selective laser sintering (SLS) is a high-speed AM technology popular for metals and polymers processing^21–23^. Successful SLS requires powders with good flowing properties, made of particles that melt together when heated for short time. Thermoset polymers are thus poor candidates for manufacturing 3D structures with this technology, with different strategies being currently proposed to improve their SLS printability^24,25^. Surprisingly though, one of the first works on AM of carbon materials relied on this technology to sinter carbon fibre/phenol-formaldehyde (PF) resin particles^26^. This approach was mimicking the sintering of carbon fibre/polyamide powders^27^, which are nowadays very popular for SLS printing of composites. According to the authors of the study^26^, the PF resin used softens/melts at 102 ºC and cures at higher temperatures. In order to maintain the integrity of the 3D printed structures, a tight control of a lengthy (20 h) carbonisation process was necessary.

This work shows the possibility of using an alternative carbon precursor, whey powder, which can be SLS printed and then readily carbonised without losing its original shape. Recently, we have described the carbonisation of whey powder (600–1000 ºC)^28^. From a Carbon Science perspective, it was actually an unexpected result to discover that whey powder does not melt when heating under N_2_at temperatures above 200 ºC, thus behaving as a thermosetting precursor. Moreover, rather than melting, whey powder particles sinter at relatively low temperatures (120–150 ºC). 3D carbon structures were then crafted by simply pouring whey powder in moulds and carbonising them. This triggered the possibility of using whey powder in a SLS printer to obtain 3D structures with complex geometries that could be easily transformed into carbon, which is the subject of the present contribution. As for the potential applications of these 3D printed whey-derived porous carbons, it is envisaged their use in tissue engineering and chemical and biochemical process intensification^29–31^.

## Results

### SLS of whey powder

**Fig. 1:**
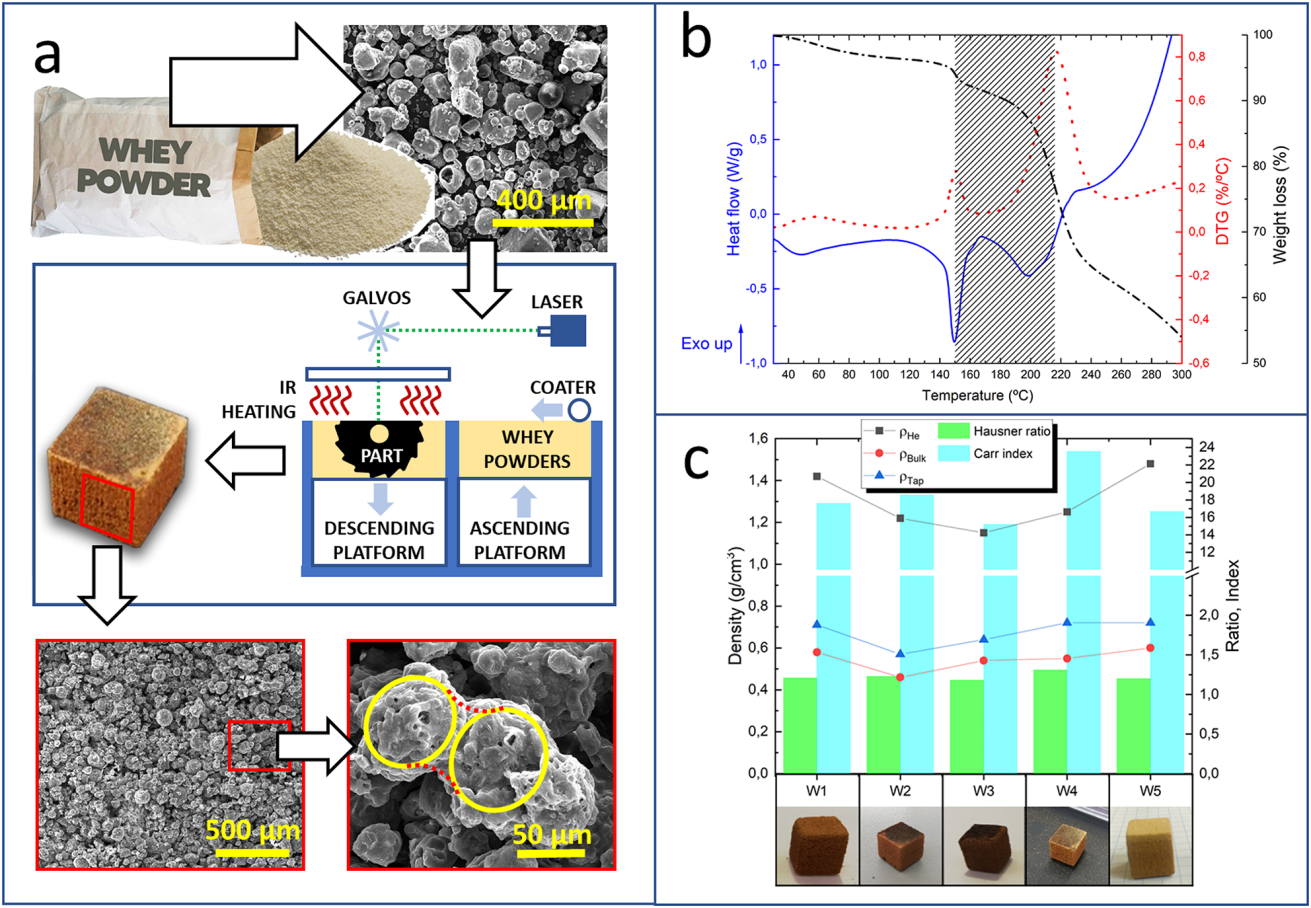
Key factors of whey powder for its selective laser sintering (SLS); (**a**) schematics of the sintering process with micrographs showing the rounded shape of the fresh whey powder and the bridging of individual whey particles after laser sintering; (**b**) TA, DTA and DSC of whey powder (W1) in air, with the SLS processing temperature window highlighted; (**c**) densities (real, bulk and tap densities) and flowability properties (Hausner ratio and Carr index) of the different whey samples (W1-W5), with SLSed cubes obtained from all of them using similar processing conditions (bed temp = 80 ºC; surface (printing) temp = 135 ºC; perimeter number = 1; perimeter offset = 75 μm; hatch offset = 75 μm; hatch distance = 75 μm; layer height = 100 μm; printing speed = 250 mm/s).

Figure 1a shows a scheme of the SLS processing of whey powder. This by-product of the dairy industry^28^ is made of particles with typical carbohydrate (mainly lactose) and protein (mainly β-lactoglobulin) contents of 65–80% and 10–15%, respectively (Table S1). Crystalline α-lactose monohydrate constitutes 80–95% of the lactose in non-hygroscopic whey powder^32^. Most whey particles are rounded, either spherical or potato-shaped, which is convenient for SLS sintering^21,22,33^. Whey particles size distribution (PSD) depend on the specific processing and origin of the liquid whey, with typical D_50_ values of 70–300 μm (Table S1). Whey powder does not completely melt in the SLS process. Instead, necking of whey particles is ubiquitous in the resulting (macro)porous structures (Fig. 1a). This effect makes possible the laser printing of 3D whey structures that preserve their shape after cooling down the powder bed and de-powdering. The non-enzymatic browning of the laser sintered whey pieces is also obvious.

The temperature window of SLS printability is defined by the DSC profile of the whey powder (Fig. 1b). The first thermal event at temperatures ca. 50 ºC is related to the amorphous lactose glass transition (T_g_) temperature, which depends on the origin and relative humidity of the whey powder^34,35^. This event was not identified in all the whey powder samples tested, which is an indicative of whey powder samples with high (> 95%) crystalline lactose contents. The endotherm peak at ca. 150 ºC corresponds to the release of the water of crystallisation of the α-lactose monohydrate present in whey^36^, and marks the lowest temperature limit of the processing of whey powder with this technique. The next wide endothermal band allows the SLS of whey powder in air up to ca. 220 ºC, when whey powder combustion would eventually start. We anticipate that higher printing temperatures would be feasible if working under inert atmosphere.

We have tested the SLS of 5 whey powder samples (W1-W5, Table S1) from diverse origin, including sweet and acid whey powder. The values of the true density (ρ_He_) of the whey powder range from 1.15 to 1.48 g/cm^3^ (Fig. 1c). Whey powder is denser than PA12 powders (1.02 g/cm^3^), whereas the ρ_He_ values of W1 and W5 are very similar to those of cured RF resin xerogels (1.41–1.44 g/cm^3^)^37^. The studied whey powder samples present different compactability and flowability as determined by their bulk (ρ_Bulk_) and tap (ρ_Tap_) densities, and the corresponding Hausner ratio and Carr index (Fig. 1c). All whey powder samples except W3 present Carr indexes > 15, the maximum expected value for a powder to have a good flowability. Moreover, W4 shows a Carr index > 21, which is considered an indicative of passable (just before poor) flowability^38^. Nonetheless, the Hausner ratios of almost all whey powder samples correspond to the critical value (1.2) that guarantees the minimum powder viscosity of polymers^39^. Only W4 has a Hausner ratio significantly higher (1.31), although a minimal increase of powder viscosity would be expected^39^. In practice, the limitations to whey powder SLS coming from its flowability properties were negligible, and SLSed cubes were obtained from all whey samples tested, under similar operational conditions (Fig. 1c). Using acid whey powder for SLS is especially relevant from the valorisation standpoint of this type of whey^40,41^.

**Fig. 2:**
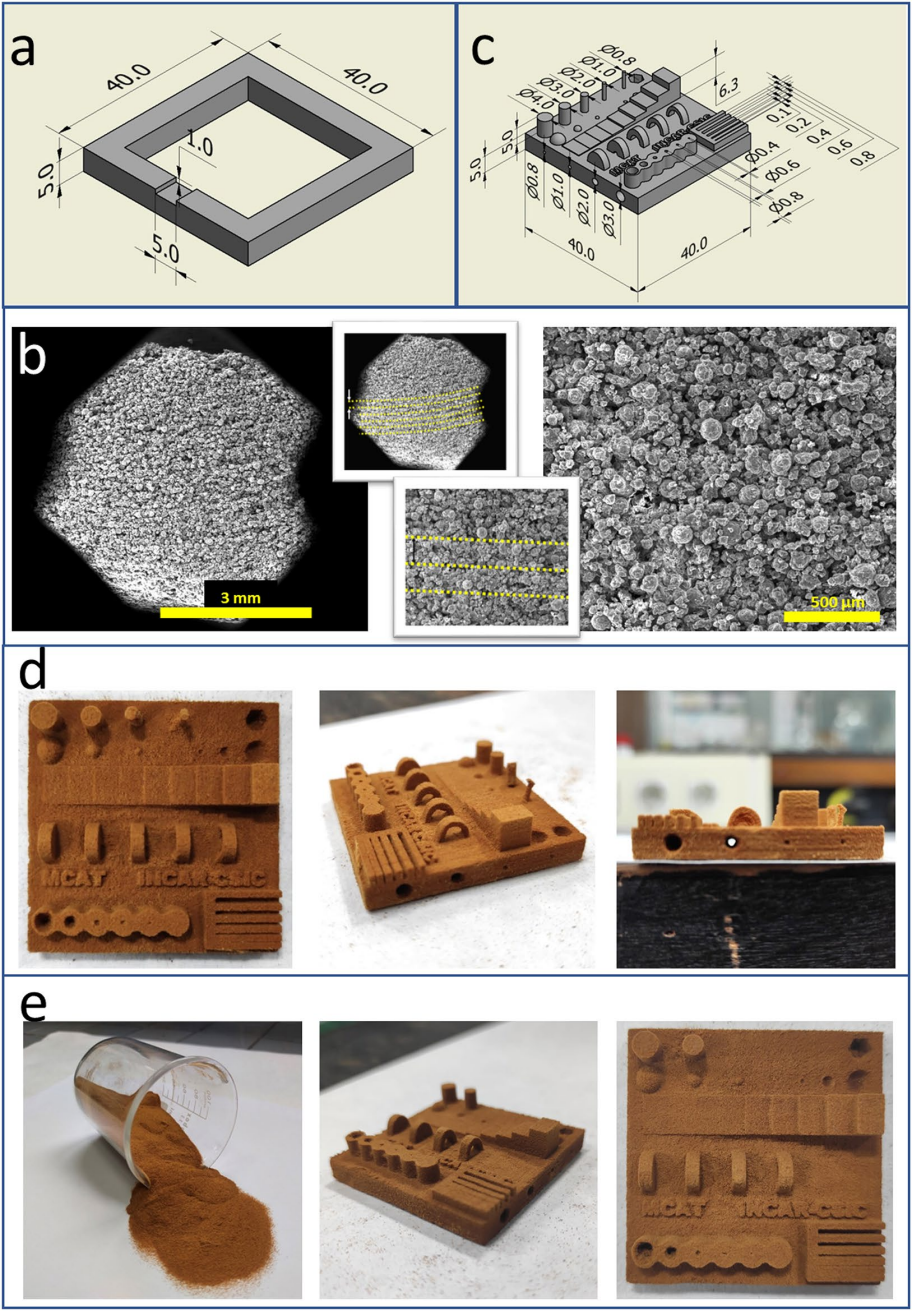
Characteristics of the selective laser sintering (SLS) of whey powder; (a) details of a simple model used for the optimisation of the printing parameters of whey powder (see Table 1); all distances are in mm; (b) micrographs of cross-sections of SLSed whey powder showing an almost imperceptible layering effect; distances between the (hardly-visible) layers are approx. 250 μm; (c) artifact test designed for testing the SLS printing precision of whey powder; all distances are in mm; (d) example of a 3D printed artifact test made of whey powder (W5); (e) photographs of the whey powder collected from the printing reservoirs after a conventional print work (3–5 h), and sieved (< 150 μm); the two other photographs correspond to a 3D printed artifact test made of this re-used whey powder. SLS conditions: particle size < 150 μm; bed temp = 80 ºC; surface (printing) temp = 135 ºC; perimeter number = 1; perimeter offset = 75 μm; hatch offset = 75 μm; hatch distance = 75 μm; layer height = 100 μm; printing speed = 100 mm/s.

Optimisation of the operational conditions to determine the SLS printing precision of whey powders was carried out with W5. A simple model (Fig. 2a) was selected for this purpose. Two qualitative printing responses were considered for the optimisation, namely the sturdiness of the sintered piece and the easiness of depowdering. Once the piece was successfully SLSed, the dimensional accuracy of the printed model was determined. A very elemental printer equipped with a low power (2.3 W) blue (445 nm) laser was used in this work. Hence, only basic SLS parameters could be optimised and the main results of the optimisation are summarised in Table 1. Essentially, the printing speed is the variable showing a higher effect in the SLS of whey powder. Neither the printing surface temperature nor the PSD of the whey powder are so relevant within the variable range explored. The latter variable (PSD) affects though to the definition of the perimeters of the printed structure, with lower PSD bringing about surfaces with better finish and sharper details. The resulting pieces have dimensional deviations < 3% of the nominal values, with no significant warpage or cracking, and easy handling overall. SEM analysis of the cross-sections of the printed models (Fig. 2b) shows a very uniform porous structure with almost imperceptible layering^42^, which highlights the homogeneous sintering of the whey powder in the z direction.

**Table 1:**
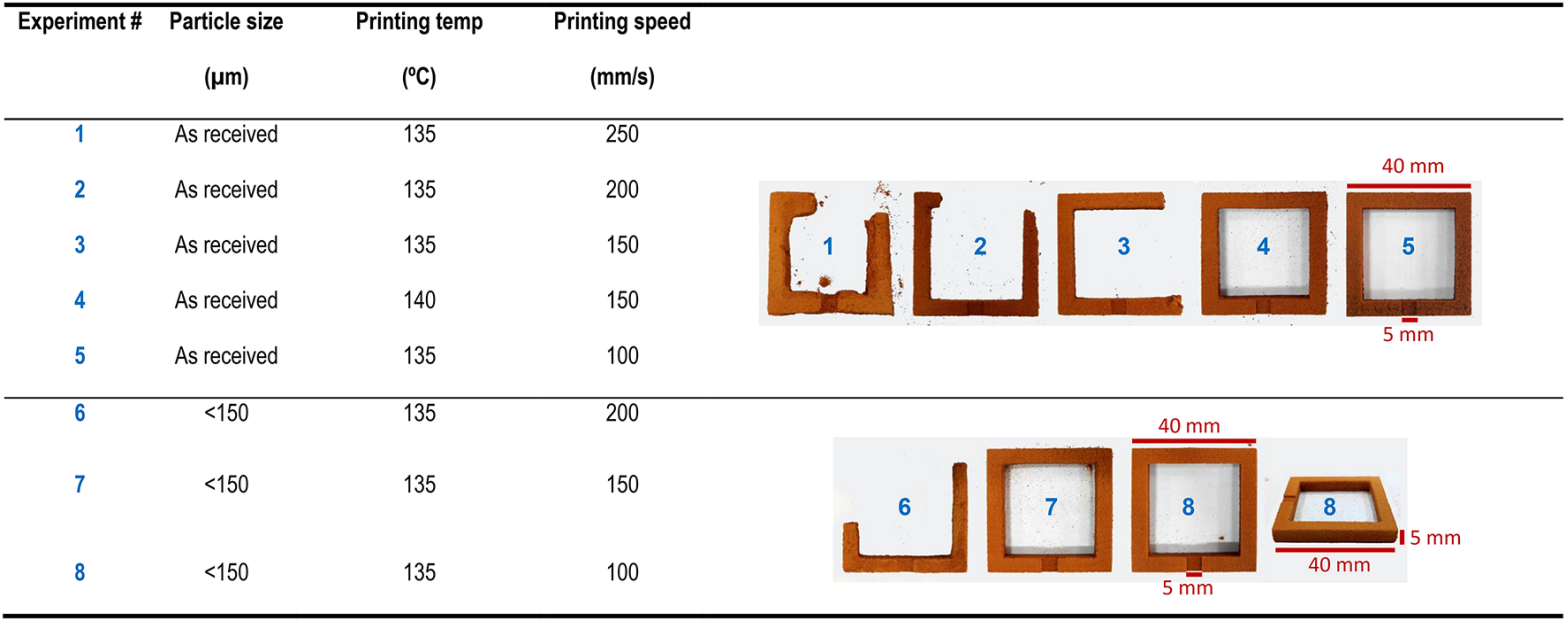
Optimisation of the SLS conditions of whey powder (W5). The model used for the optimisation is depicted in Fig. 2a. Numbers in the photographs correspond to Experiment #.

The printing conditions of experiment 8 were then selected for further studies. An artifact test (Fig. 2c) was designed to determine the SLS printing precision of whey powder. Figure 2d shows an example of the printed test, which includes rods, bulges, grooves, surface and through-holes, bridges and steps of different dimensions. Most features are present in the sintered whey structure, whereas absent or poorly defined elements are either related to the post-processing (brushing off the slenderer rods is quite challenging) or to the limitations of the whey powder sintering (i.e., through-holes < 2 mm).

Whey reuse was also contemplated in this study, and results are shown in Fig. 2e. In spite of the evident browning and caking of the spare whey powder remaining in the two printer reservoirs, a simple sieving (< 150 μm) procedure brings about a powder with adequate flowability and sintering behaviour to be successfully re-processed. Similar results were also obtained with powders processed for a second time, i.e., after two heating and cooling down cycles in the SLS printer.

### From SLS printed whey powder to complex 3D pieces made of porous carbon

**Fig. 3:**
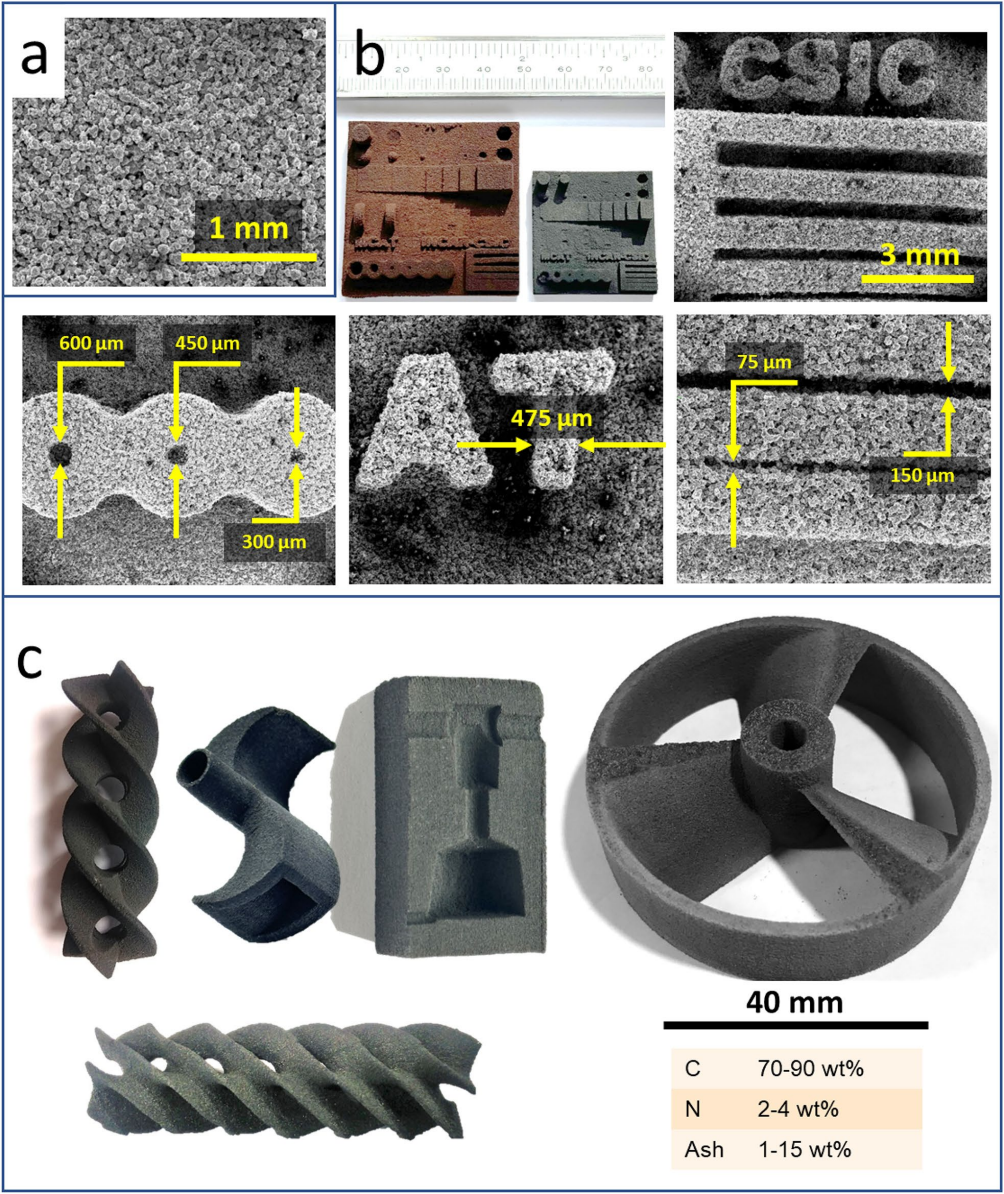
Porous carbon materials from SLSed whey powders; (a) micrograph of a cross-section of a carbonised SLSed structure, in which the rounded particles of the fresh whey (Fig. 1a) and sintered whey powder (Fig. 2b) are still clearly visible, with no layering at all; (b) carbonisation (850 ºC, 1.5 h) preserves the original form of SLSed whey powder structures, but with a significant (23%) isotropic contraction; accordingly, all the distances in the porous carbon artifact test (Fig. 2c) are reduced in approx. 23%, as shown in the different micrographs, reaching an outstanding printing precision; (c) examples of porous carbon with complex 3D structures (two fusilli-like helicoids, a Ugrinsky wind turbine, the aluminothermic mould and a pitch-blade turbine), presented as a proof of concept; typical ranges of C, N and ash contents (wt%, dry basis) of the SLS whey-derived carbons, depending on the specific whey powder used and/or porous carbon modification (e.g., acid washing). Whey powder SLS conditions: same as those detailed in Fig. 2.

Once the whey powder is sintered, transformation into a porous carbon is straightforward as already described^28^. A SEM image of the cross-section of a SLS whey derived carbon (Fig. 3a) shows a homogeneous porous structure made of carbonised sintered whey particles. The similarities between Figs. 2b and 3a confirm the thermosetting nature of the sintered whey, which does not melt during the carbonisation treatment. As a result, any 3D structure made of sintered whey does not deform during carbonisation and all the features present in the original are preserved albeit a 23% isotropic shrinkage (Fig. 3b). The attainable resolution of the resulting SLS printed porous carbon structure is astonishing, comparable to that of carbons prepared by SLA of photocurable resins^8–11^. Figure 3c shows some examples of 3D structures made of whey-derived porous carbon. They are presented here as a proof of concept of the capabilities of the methodology. Some of those structures with intricate overhangs cannot be produced with other conventional AM technologies such as FDM or DIW. Typical manufacturing times are 6–8 h, i.e., 3–5 h of SLS printing and de-powdering, plus 3 h carbonisation (at 850 ºC, 1.5 h dwell time).

**Fig. 4:**
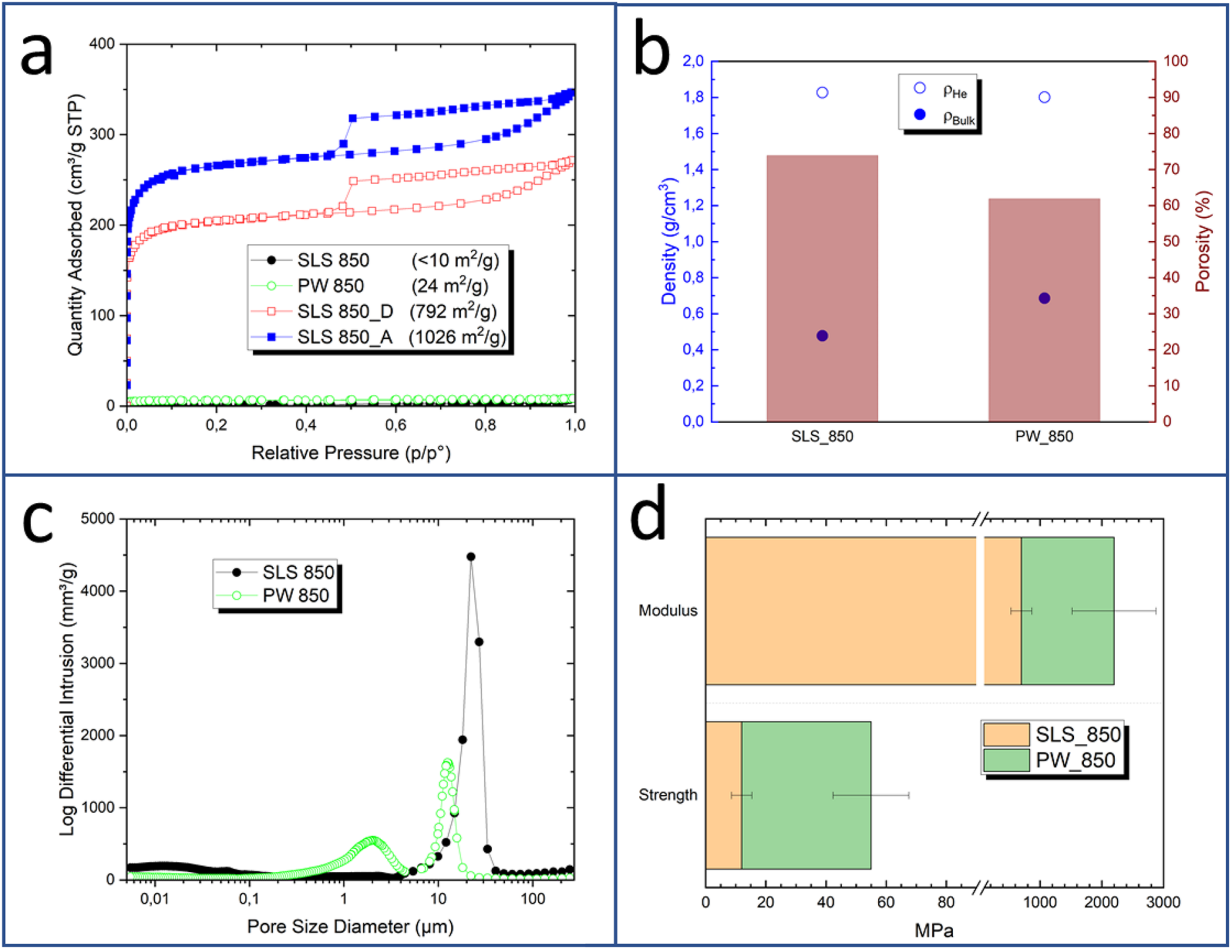
Porous and mechanical properties of SLSed whey-derived carbons (carbonised at 850 ºC, 1.5 h, SLS 850), and comparison with moulded whey-derived carbons (carbonised at 850 ºC, 1.5 h, PW 850); (a) N_2_ adsorption isotherms (-196 ºC); labelling: SLS 850_D, demineralised carbon; SLS 850_A, thermally (CO_2_) activated carbon; (b) densities and porosities of sintered (SLS 850) and moulded (PW 850) whey-derived porous carbons; (c) Pore Size Distributions (PSD) of the SLSed (SLS 850) and moulded (PW 850) porous carbons, as measured by Hg intrusion; (d) compressive strength and modulus of SLSed (SLS 850) and moulded (PW 850) whey-derived porous carbons.

As for the properties of the SLSed porous carbons, they have similarities with those obtained by moulding^28^ (Fig. 4). Thus, typical elemental composition analysis indicates C and N contents of ca. 75 wt% and 3 wt%, respectively (Fig. 3c). This relatively high N content of the 3D carbon materials is interesting for electrochemical and catalytic applications^44,45^. Whey powder-derived carbons have an initial ash content of 10–13 wt%. The mineral matter in these porous carbons comes from the soluble salts present in the liquid whey after dairy processing. Figure S1shows an XRD profile of the SLS 850 porous carbon. In addition to the wide band centred at ca. 22º 2θ that correspond to the poorly crystalline carbon phase, additional peaks come from the mineral salts, mainly chlorides and phosphates of alkaline and alkaline earth elements. Almost half of the mineral matter in the carbons is soluble in water. Furthermore, washing with HCl removes it to levels < 3 wt%^46^. Another similarity of SLSed and moulded 3D carbon structures is that they adsorb little to none quantities of N_2_ at cryogenic temperatures (Fig. 4a). This changes when the mineral matter is washed off with HCl (sample SLS 850_D), rendering a carbon material with a relatively high surface area, which can be further increased by thermal activation procedures (sample SLS 850_A) (Fig. 4a)^46^.

Dissimilarities between porous carbon materials obtained by SLS or moulding and further carbonisation of whey powder are, however, relevant in terms of the overall porosity of the resulting structures. The bulk density of the carbon pieces obtained by SLSed carbons is considerably lower than that of moulded whey powder-carbons (Fig. 4b). Accordingly, the porosity of the SLSed structures is as high as 74%. The macropore size distributions of the SLSed whey-derived carbons shift to higher pore sizes when compared to the moulded ones (Fig. 4c), this being the reason for the increase of the porosity from 60 to 74%. The occurrence of wider macropores on the SLS printed carbons also affects the mechanical properties of the 3D structures, with a significant decrease of their compressive strength and modulus (Fig. 4d). Still, the compressive performance of the SLSed carbon materials is very good considering their high porosity^46^.

## Discussion

The processing of whey powder with SLS is related to previous studies using the same technique for 3D printing of carbohydrates. Thus, SLS of glucose and sucrose^47^, and isomalt^48^has been already reported for biomedical applications. Also, even though SLS of pure lactose has not been reported, drug formulations including quantities of α-lactose monohydrate (up to a 12 wt%) have been SLSed for printlets manufacturing^49^. According to the different researchers, the fabrication of the 3D printed structures from these powders involves melting/caramelisation of the sugars. However, the presence of proteins in whey powder makes a substantial difference in the sintering of whey particles. Furthermore, that very same concomitance of lactose and proteins in whey is compulsory to preserve the SLS printed shape during carbonisation^28^.

There is scarce information on the particle sintering of dairy powders at temperatures above 100 ºC. Three possible mechanisms are postulated here to be, in principle, responsible for the sintering of whey particles, namely caking, caramelisation and Maillard reactions. The two first are specific of lactose, the main constituent in whey, and the latter requires the coexistence of lactose and proteins. The sintering of whey powder depends on the temperature/time combination, and sintered structures can be obtained from 85 ºC on if enough time (at least 2 h) is given (Figure S2). On the other hand, much higher temperatures are required when the heating times (2 min) come close to the conditions of the SLS processing (Figure S3). At such short times, the lump obtained at 160 ºC was easily friable (Figure S3); whereas at 220 ºC, 2 min, the whey powder started to scorch, as predicted from Fig. 1b. In all cases, non-enzymatic browning is evident, thus pointing out the occurrence of pigments characteristic of Maillard reactions and/or lactose caramelisation.

Caking (i.e., particle sintering) of lactose is well studied for its implications in the preservation of dairy powders and pharmaceutics^50^. The main mechanisms of lactose caking at temperatures well below 100 ºC include amorphous, humidity and mechanical caking^35,51^, and the particle bridging observed in Fig. 1a resembles that depicted in micrographs of caked whey powder^52^. However, the SLS conditions used in this work are far from those required for whey (lactose) caking, namely high amorphous lactose contents and/or high water activities in the whey powder^32,35,50–52^. As shown in Video S1, α-lactose monohydrate powders cake to a certain extent after 2 h at 160 ºC, whereas 2 min at such temperature has no apparent effect on powder compaction (Figure S4).

Lactose caramelisation is known to happen at temperatures close to 220 ºC (Figure S5)^53,54^. It involves the pyrolytic breakage and further polymerisation of the galactose and glucose molecules of lactose, and the physical transformation of the powder into a viscous glass^55^. This has obvious implications in the SLS of whey particles, which would stick together in a similar fashion to other SLSed carbohydrates, as mentioned above. Experimentally though, caramelisation of lactose requires relatively long times at high temperatures to be effective (Figure S6). When approaching to the sintering conditions on the SLS printer, i.e., 2 min at 200º C, α-lactose monohydrate powders remain loose and white (Figure S7).

Maillard reactions between lactose and whey proteins are responsible of the non-enzymatic browning of dairy powders at ambient temperature^55^. Whereas Maillard reactions and caking (particle sintering) are identified as damages occurring during storage of whey powders^56,57^, a possible contribution of Maillard reaction products (e.g., melanoidins) to whey particle sintering has not been established. Melanoidins are brown coloured polymers formed in the so-called late-phase of the Maillard reactions^58,59^. In spite of a detailed structural characterisation, melanoidins are roughly classified attending to their size into low, intermediate and high molecular weight, with their molecular weight distribution (MWD) depending on a number of factors including temperature/time conditions of the reaction. It is now accepted that low molecular weight (1–10 kDa) melanoidins in reducing sugars/protein systems (e.g., whey) are chromophore aggregates covalently bonded to protein backbones^60,61^. These aggregates account for the colour of the melanoidins and come from the dehydration and condensation of the carbohydrates. Heating up the reducing sugars/protein systems shifts the MWD of melanoidins towards higher values, reaching values > 100 kDa^59–61^. High molecular weight melanoidins include multiple protein backbones crosslinked with the chromophores, which, in addition, are known to polymerise with temperature^60,61^. It is also accepted that carbohydrate caramelisation products contribute in Maillard reactions at high temperatures^62^. Furthermore, characterisation of melanoidins in roasted malts demonstrates that there is a temperature threshold (ca. 160 ºC) for the formation of high molecular size melanoidins^63^.

This melanoidin size-increase pattern in Maillard reactions resembles the curing mechanism of PF or RF polymeric resins, and it is postulated responsible for the sintering of whey powder with temperature. Thus, the sintering mechanism proposed here involves the progressive enlargement with temperature of melanoidins on the surface of the whey particles. It should be pointed out that the relative lactose/whey protein ratio concentration is considerably lower at the outermost surface of the whey particles^64^, thus making this mechanism especially relevant. The completion of the melanoidins “curing” process, i.e., the maximisation of their MWD, would be required to attain a strong inter-particle bond that endures carbonisation and keeps the shape of the SLSed whey structure unaltered.

The final part of this discussion is devoted to explain the lower density of the SLSed whey derived carbons when compared to the moulded ones. We found this to happen regardless the origin/type of the whey powder. This result was unexpected in light of the minimum layering effect observed in the SLS process (Figs. 2b and 3a). Pieces of W5 were then SLSed using a higher printing layer height (200 μm) to check if this parameter could have an effect on the density of the resulting carbons, but their bulk density (0.44 g/cm^3^) was the same than those obtained when using 100 μm printing layer height (SLS_850 in Fig. 4b). At this point of the discussion, It should be clear that whereas fresh whey powders are used in the moulding process, SLS of whey powder does not actually sinter fresh powder, as it browns (Maillard reaction) due to the heating required to reach and maintain the bed and printing temperatures (80 ºC and 135 ºC, respectively) for approx. 4 h. Moreover, a very relevant result was found when re-used whey powders (i.e., not fresh whey powder, Fig. 2e) were poured in a mould and carbonised at 850 ºC (1.5 h). The bulk density of the resulting carbons was 0.48 g/cm^3^, very similar to that of the SLSed whey derived carbons. In other words, it is not moulding or SLS of whey powders what would make the difference in the density and mechanical properties of the resulting carbons. Under the same carbonisation conditions, sintering of fresh whey powders brings about denser carbons than sintering brown whey powders.

As mentioned above, the brown whey powder is lightly sintered after the printing process. The browning process has little effect on the SLS processability parameters of the whey powder (Figure S8), but changes significantly its TG and DSC profiles (Figure S9). Specifically, the loss of crystalline water at ca. 150 ºC is much lower in the brown powder. This is expected and denotes the transformation of the α-lactose monohydrate present in the original whey powder into different polymorphs when dehydrated at high temperature (> 120 ºC)^65–67^. Specifically, the loss of crystalline water at such temperature leads to two anhydrous α-lactose phases, one unstable (hygroscopic) and the other stable, and to anhydrous β-lactose, with the two latter polymorphs prone to cake after short times^67^. Actually, this transformation explains the caking of the lactose powders shown in Video S2. Furthermore, the caked lactose after dehydration is friable, a similar behaviour observed for the brown whey powder.

Since we do not expect different lactose polymorphs to affect the Maillard reactions products significantly, there are two possible explanations for the lower density of the brown whey derived carbons. The first one would imply the sintering of the fine particles, thus affecting the PSD of the browned whey. This was discarded when considering the flowability parameters (Figure S8) and by measuring the PSD (Figure S10) of the fresh and re-used W5 powders. The other, more plausible possibility is related to the behaviour of the melanoidins with temperature once they are formed at a given temperature and then cooled down. We have observed that if, after cooling down, we break a 3D structure of whey sintered in a mould at a given temperature (e.g., 120 ºC, 2 h) and try to stick the two parts together by re-heating them, it is not possible. This, again, is a similar behaviour of conventional thermoset resins. As a consequence, the whey interparticle bridging attained at a given temperature cannot be reconstructed if broken, thus reducing the contact points between whey particles and hence lowering the density of the sintered pieces.

## Methods

Whey powder of different companies was used in this study, namely, LaFuente (W1), Lemasa (W2 and W3), CAPSA FOOD (W4) and Lactalis (W5). Table S1 collects details of their composition, according to the supplier specifications. α-lactose monohydrate (> 99.5%) powder from MERCK was used for reference studies.

Selective Laser Sintering (SLS) of the whey powder was carried out in a Sintratec Kit bench printer equipped with a low power (2.3 W) blue (445 nm) laser. According to the manufacturer specifications, the laser spot is 50 μm in diameter. The printer is controlled by proprietary software that allows to control a few processing parameters, namely surface (printing) and chamber (bed) temperatures, printing speed, layer height, perimeter number, perimeter offset, hatch offset and hatch distance. Chamber (bed) temperature, perimeter number, perimeter offset, hatch offset and hatch distance were fixed in all experiments at 80 ºC, 1, 75 μm, 75 μm and 75 μm, respectively. Layer height was set at 100 μm for all experiments except for the study of the effect of whey powder packing (layer height of 200 μm). Surface (printing) temperature and speed were initially set at 135 ºC and 250 mm/s. These were the printing conditions used for the sintering of the whey cubes in Fig. 1c. Optimisation of the surface (printing) temperature and speed was then carried out as explained in the text. The whey powder in the reservoir is conditioned at the chamber (bed) temperature for approx. 45 min before the laser starts to print. The surface (printing) temperature is set and maintained during approx. the last 30 min of the chamber conditioning. Once the printed work is finished, the chamber (bed) temperature is maintained for 30 min before cooling down. Depowdering of the printed structures was done manually by brushing-off the non-sintered powder. The SLSed whey parts were then transferred to a tubular oven (Carbolite) and heated up to the desired temperature (850 ºC), at 10 ºC/min under 150 ml/min of N_2_. Dwell time at 850 ºC was 1.5 h. Video S1 in Supplementary Information shows a synopsis of the whole process. The STL files for the manufacturing of the artifact test (Fig. 2c), the Ugrinsky wind turbine, the aluminothermic mould and one of the fusilli-like helicoid presented in Fig. 3c are available at^68^. All these pieces were designed using Autodesk Inventor Professional 2015 (USA). The rest of 3D objects (the long fusilli-like helicoid, pitched-blade turbine and Big Ben replica) are designs available in the web.

Additional treatments to the porous carbons obtained from the SLS of whey powder include acid washing and thermal (CO_2_) activation. The acid washing protocol includes four cycles of 15 min into sonicated water at 80 °C, replacing the washing water after each cycle. Then, the structures were sonicated in a 37 wt% HCl solution for four cycles of 15 min. Finally, they underwent four cycles of sonication (15 min) with distilled water at 80 °C to remove the acid residues. For CO_2_ activation, the demineralised structures were heated at 850 °C (10 ºC/min) under 150 ml/min of N_2_. When reaching the set temperature, the N_2_ flux was switched to 50 ml/min of CO_2_ for 1 h. The activated structures were cooled down in N_2_ (150 ml/min).

Characterisation of the whey powder and porous carbons include DSC in air and inert (N_2_) atmospheres. Experiments were carried out in a DSC 2010 calorimeter (TA Instruments) under 50 ml/min of air or N_2_. 25 (± 0.1) mg of whey powder were placed in closed aluminium pans and 5 ºC/min heating ramps were used to reach the set temperature. Similar conditions but with open ceramic pans were used in the TA/DTA experiments (SDT Q600 thermobalance, TA Instruments).

Bulk densities (ρ_Bulk_) of the whey powders were measured by pouring a given mass (60 g) into a 100 ml test tube and checking the powder filling volume. Tap densities (ρ_Tap_) of the whey powders were then manually measured using the same 100 ml test tube in accordance with ISO 23145-1:2007, i.e., by subjecting the tube to 1000 taps in 10 min and checking again the filling volume. Whey powders flowability and compressibility parameters such as Hausner ratios and Carr indexes, respectively, were calculated from the corresponding ρ_Bulk_ and ρ_Tap_ as follows: Hausner ratio = ρ_Tap_/ρ_Bulk_; Carr index = [1-(ρ_Bulk_/ρ_Tap_)]×100. Whey powder particle size distributions (PSD) were determined in a LS 13 320 Laser Diffraction Particle Size Analyser (Beckman Coulter) equipped with a Tornado dry powder system. True (ρ_He_) and apparent (ρ_Hg_) densities of the whey-derived carbons were measured by He and Hg intrusion, respectively, as detailed elsewhere^28^. The porosity (%) of the carbons was then calculated as follows: Porosity = [1-(ρ_Hg_/ρ_He_)]×100. Elementary composition analyses, N_2_adsorption isotherms (-196 ºC) and Hg intrusion characterisation (i.e., pore size distributions) were also described in previous works^28,46^. SEM images were obtained in a Quanta FEG 650 microscope. Metal (Ir) coating was only required for the SEM characterisation of sintered whey powder pieces. XRD diffraction of the SLSed carbons was carried out in a D8 Advance Diffractometer (Bruker) using Cu-Kα radiation (wavelength = 0.1542 nm) working at 40 mA and 40 kV of current intensity and voltage, respectively. XRD profiles were measured from 15º-90º 2θ, with a step size of 0.02º 2θ and 3 s step time. Compression tests of the porous carbons were carried out in a SMT1-100 N device (MTS System Corporation), using a cell load of 5 kN and a test velocity of 0.5 mm/min, according to the ASTM C1424-15 Standard. The SLSed whey specimens were bulk cylinders made of 165 sintered layers. After carbonisation (850 ºC, 1.5 h) the diameter of each porous carbon cylinder (6.4 ± 0.1 mm) was the average of three measurements along the cylinder length. Length of the cylinders was 12.7 ± 0.1 mm. Porous carbon cylinders of similar dimensions made by whey powder moulding were also tested. The compressive strength value was obtained from the maximum load registered in the test. Elastic compressive modulus (E) was calculated from the slope of the linear elastic region. Compressive strengths and moduli reported are the average of three compressive tests ± standard deviation. Stress-strain curves of those tests can be found in the Supplementary Information (Figure S11).

## Electronic supplementary material

Below is the link to the electronic supplementary material.

Supplementary Material 1

## Data Availability

All data generated or analysed during this study are included in this published article [and its supplementary information files].
