# Supplementary material for: Porous carbons with complex 3D geometries via selective laser sintering of whey powder
Source: Sci Rep. 2025 Jan 13;15:1881. doi: 10.1038/s41598-024-84976-y (PMC11730622; doi:10.1038/s41598-024-84976-y)
Supplement: Supplementary file 1 — Supplementary Material 1 [file 41598_2024_84976_MOESM1_ESM.docx]

**Porous carbons with complex 3D geometries via selective laser sintering of whey powder**

Raúl Llamas-Unzueta, Alejandro Reguera-García, Miguel A. Montes-Morán*, J. Angel Menéndez

*Instituto de Ciencia y Tecnología del Carbono, INCAR-CSIC, c/Francisco Pintado Fe 26, 33011 Oviedo, Spain*

** Correspondence: miguel.montes@csic.es*

**Supplementary information**

**Table S1.** Supplier specifications of the different whey powder samples; and their particle size dsitributions.

| **Whey sample** | **Supplier** | **Type** | **Lactose (%)** | **Protein (%)** | **Ash (%)** | **Fat (%)** | **Moisture (%)** |
| --- | --- | --- | --- | --- | --- | --- | --- |
| W1 | Lafuente | Sweet | 67 | 10 | 9-15 | <1.75 | 2-5 |
| W2 | LEMASA | Acid | 62-68 | 7 | 10-14 | 0.5 | 4 |
| W3 | LEMASA | Sweet | 70 | 12 | 9 | 2 | 4 |
| W4 | CAPSA | Acid | 77 | 12.7 | 4.5 | 1.8 | 4 |
| W5 | Lactalis | Sweet | 75.5 | 11 | 8.5 | 1 | 4 |
| **Whey sample** | **D_10_**  **(µm)** | **D_50_**  **(µm)** | **D_90_**  **(µm)** |  |  |  |  |
| W1 | 36.5 | 116.8 | 237 |  |  |  |  |
| W2 | 11.1 | 80.9 | 219 |  |  |  |  |
| W3 | 26.8 | 109.5 | 260.8 |  |  |  |  |
| W4 | 19.8 | 72.7 | 139.9 |  |  |  |  |
| W5 | 97.3 | 284.3 | 590.4 |  |  |  |  |

**Video S1.** Porous carbon artifacts by SLS of whey powders.mp4. <http://hdl.handle.net/10261/356996>.

**Video S2.** lactose_caking.mp4. <http://hdl.handle.net/10261/356996>.


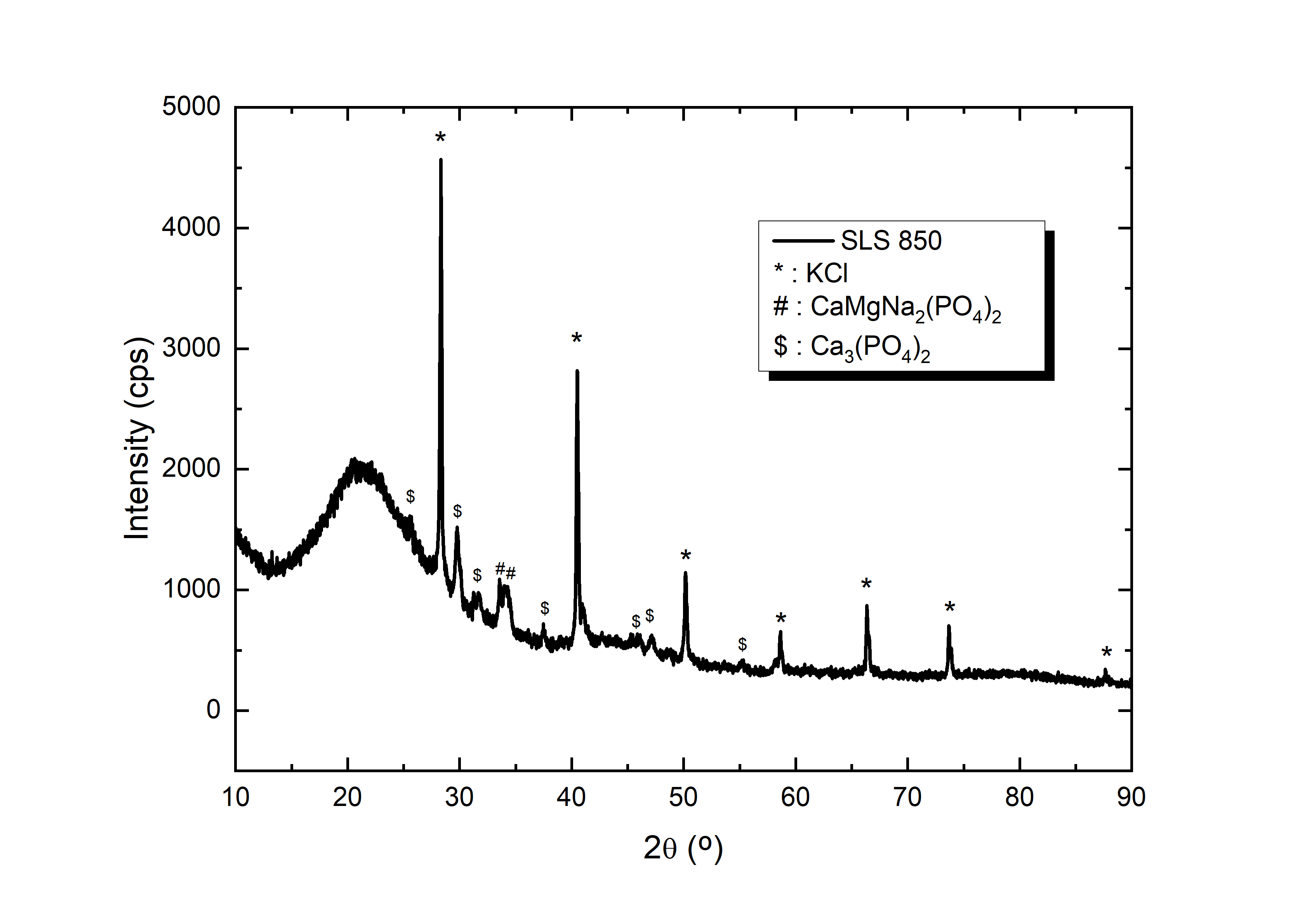


**Figure S1.** XRD profile of sample SLS 850.


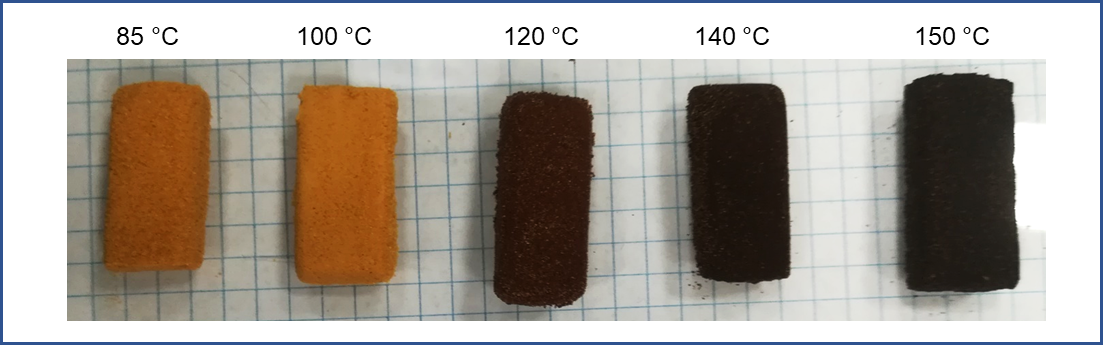


**Figure S2.** Whey powder (W5) sintered pieces heated in a mould at the indicated temperature for 2 h. Pieces sintered at 85 ºC are easily friable.


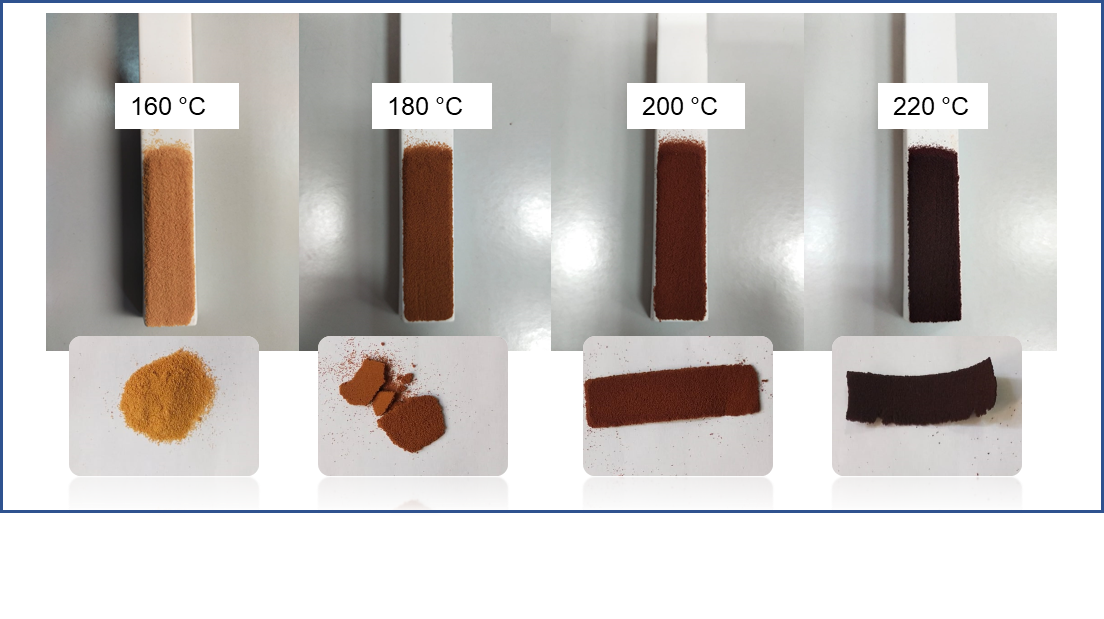


**Figure S3.** Colour and consistency of a layer of whey powder (W5) heated at the indicated temperature for 2 min.


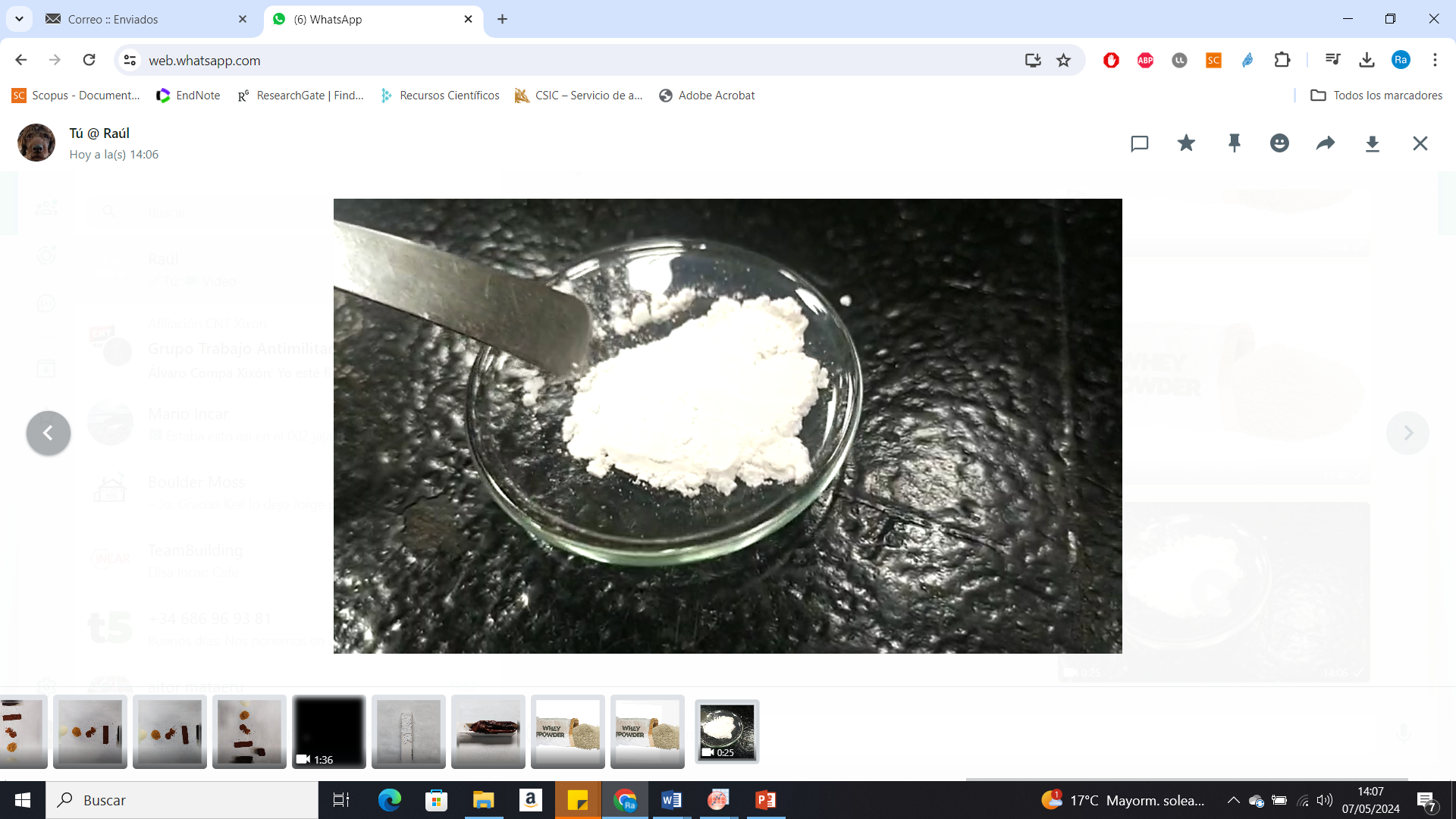


**Figure S4.** α-lactose monohydrate powders heated up to 160 °C for 2 min.


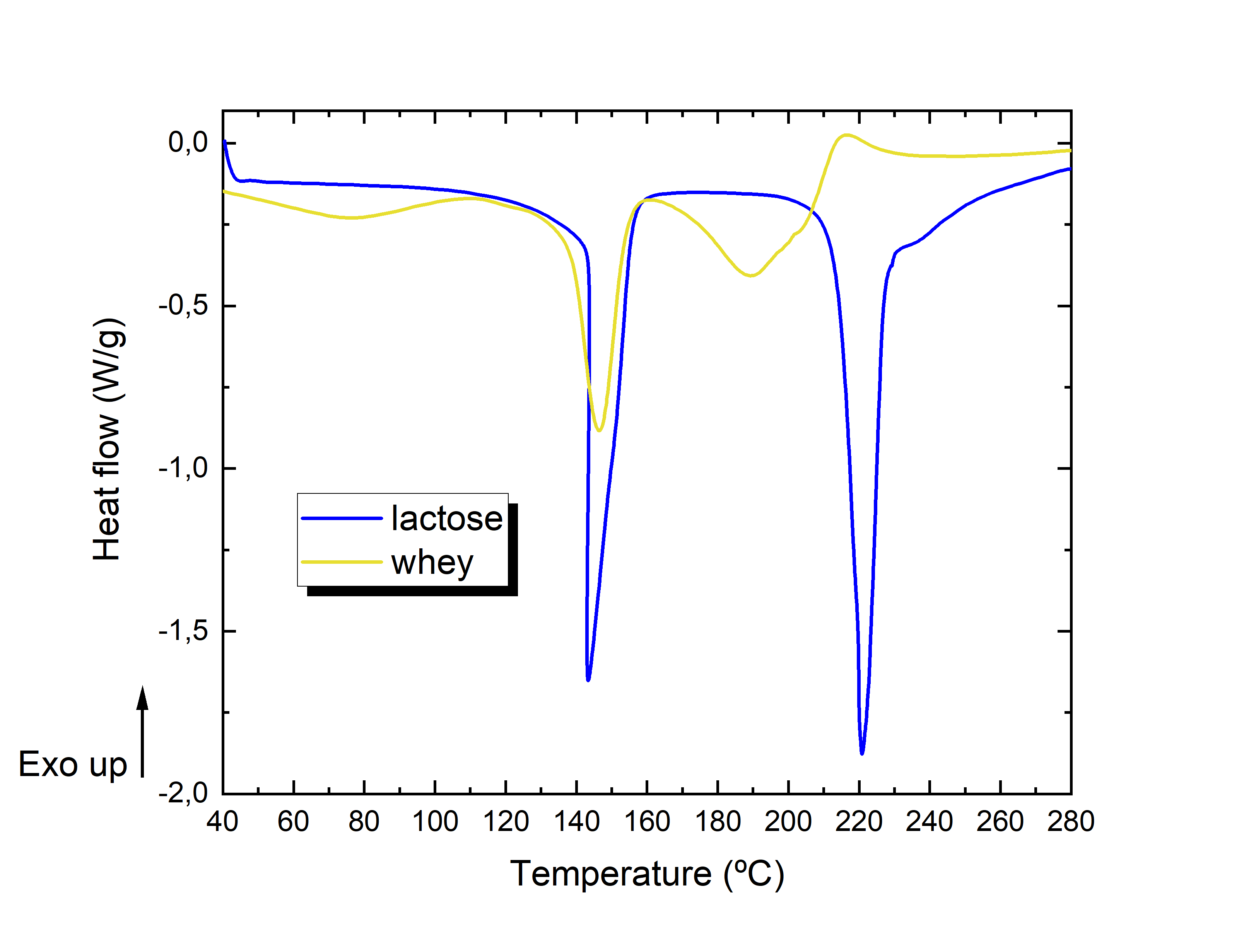


**Figure S5.** DSC curves (in N_2_) of α-lactose monohydrate and whey powder (W5).


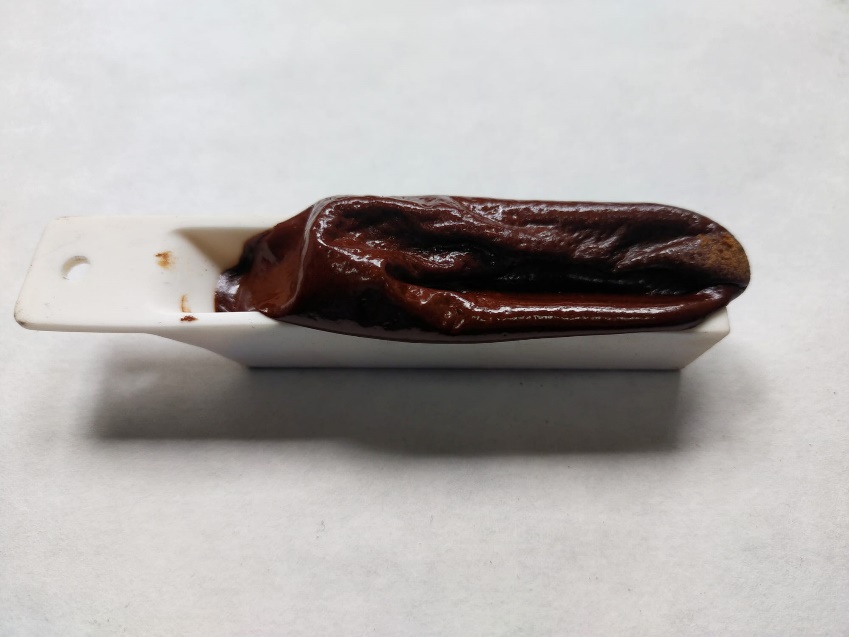


**Figures S6.** α-lactose monohydrate powder heated at 200 °C for 2 h.


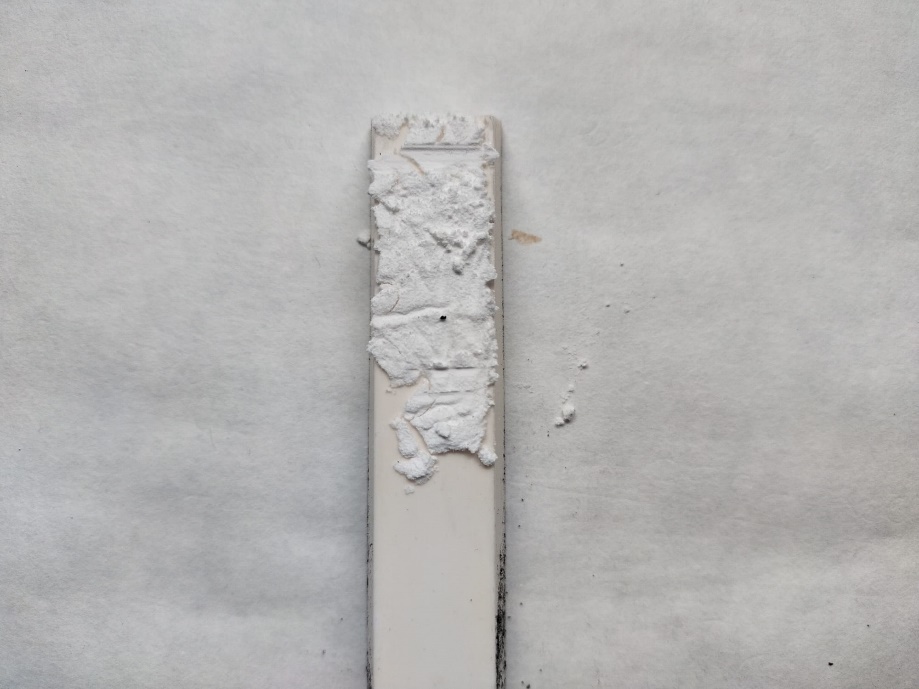


**Figure S7.** Layer of α-lactose monohydrate powder heated at 200 °C (2 min).


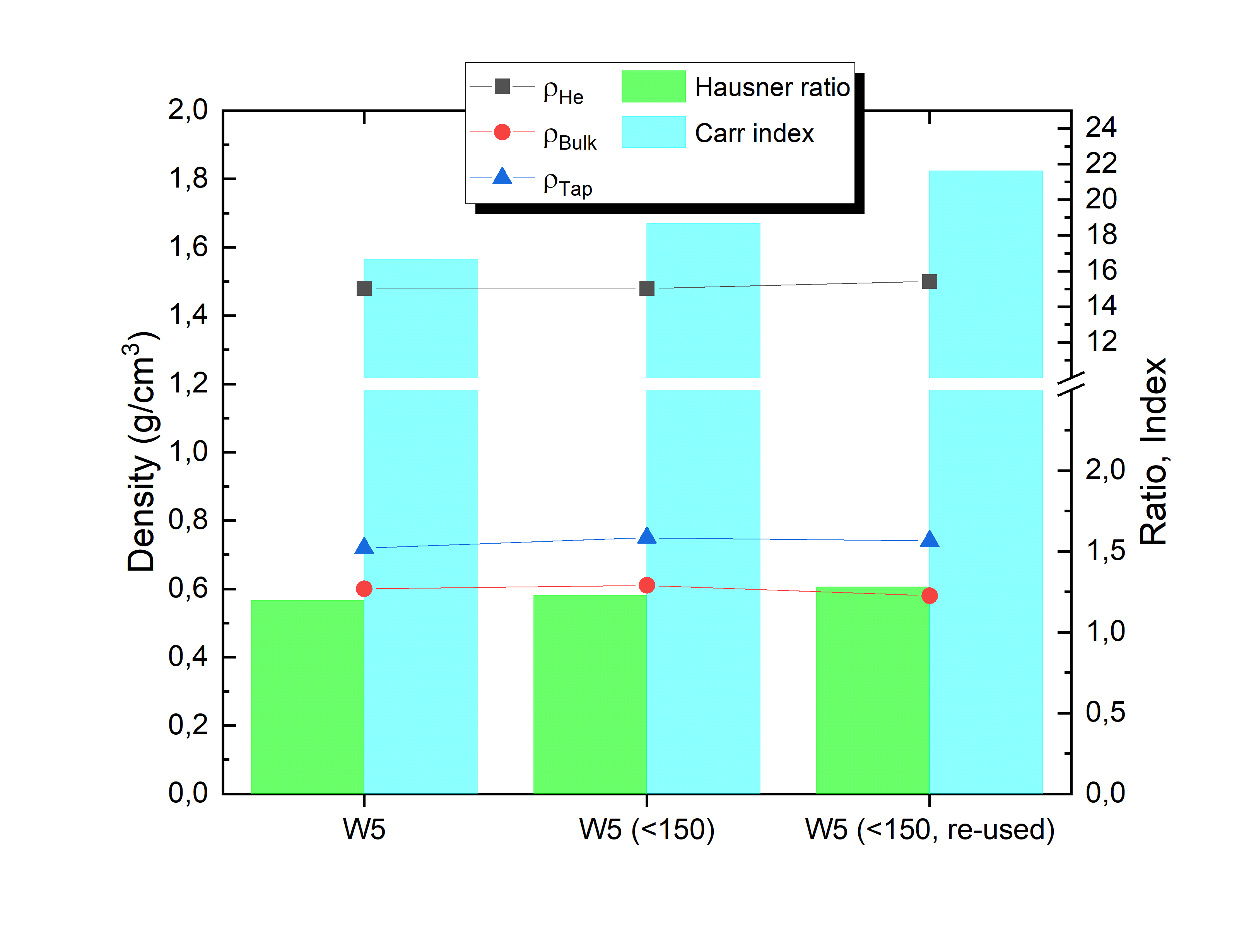


**Figure S8.** Densities and flowability parameters of: W5, as received; W5 (<150), sieved below 150 µm; and W5 (<150, re-used), sieved below 150 µm, and then re-used brown powder.


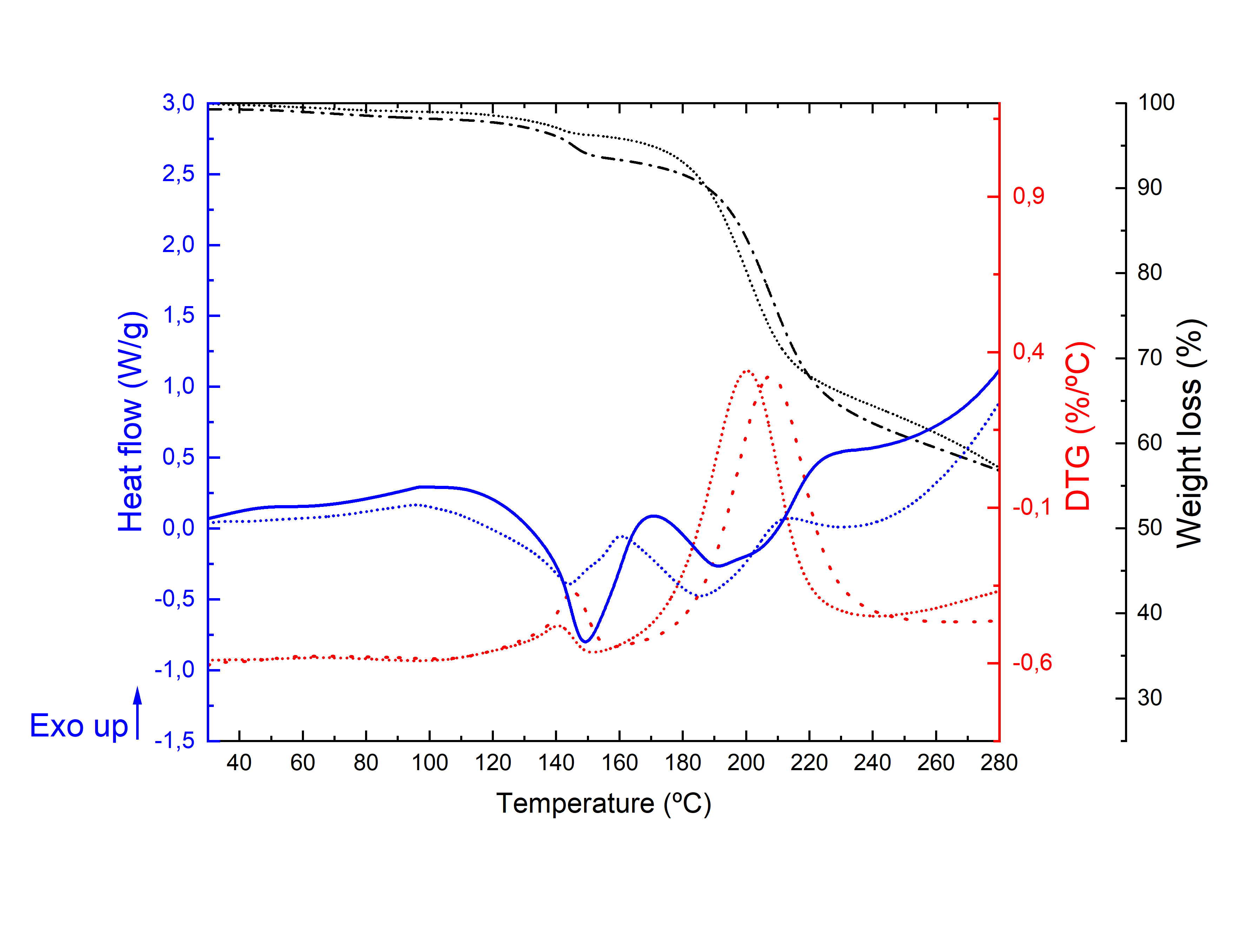


**Figure S9.** TG and DSC profiles (in air) of fresh whey powder W5 (solid blue, dotted red and discontinuous black line), and re-used brown powder (short dotted lines in every colour).

**Figure S10.** Particle size distributions of fresh whey powder W5 (red line), and re-used brown powder (blue line). D_10_, D_50_ and D_90_ values are in microns.

**Figure S11.** Stress-strain compression curves of the SLS 850 whey-derived carbons.
